# Heterogeneous Pattern of Dependence on Anti-Apoptotic BCL-2 Family Proteins upon CHOP Treatment in Diffuse Large B-Cell Lymphoma

**DOI:** 10.3390/ijms20236036

**Published:** 2019-11-30

**Authors:** Mathilde Rikje Willemijn de Jong, Myra Langendonk, Bart Reitsma, Marcel Nijland, Anke van den Berg, Emanuele Ammatuna, Lydia Visser, Tom van Meerten

**Affiliations:** 1 Department of Hematology, University Medical Center Groningen, University of Groningen, 9713 GZ Groningen, The Netherlands; m.r.w.de.jong@umcg.nl (M.R.W.d.J.); m.langendonk@umcg.nl (M.L.); bartreitsma@outlook.com (B.R.); m.nijland@umcg.nl (M.N.); e.ammatuna@umcg.nl (E.A.); 2 Department of Pathology and Medical Biology, University Medical Center Groningen, University of Groningen, 9713 GZ Groningen, the Netherlands; a.van.den.berg01@umcg.nl (A.v.d.B.); l.visser@umcg.nl (L.V.)

**Keywords:** diffuse large B-cell lymphoma, CHOP, BCL-2, MCL-1, BCL-XL, venetoclax, S63845

## Abstract

Expression of the anti-apoptotic B-cell lymphoma 2 (BCL-2) protein in patients with diffuse large B-cell lymphoma (DLBCL) strongly correlates with resistance to standard therapy with cyclophosphamide, vincristine, doxorubicin, prednisolone, and rituximab (R-CHOP). Although studies focus mainly on the contribution of BCL-2, here we also investigate the contribution of other anti-apoptotic proteins to CHOP-therapy resistance in DLBCL. Functional dynamic BCL-2 homology (BH)3 profiling was applied to DLBCL cell lines upon CHOP treatment or single CHOP compounds. Cell-specific anti-apoptotic dependencies were validated with corresponding BH3-mimetics. We found high expression of anti-apoptotic BCL-2, MCL-1, and BCL-XL in DLBCL cell lines and patients. CHOP treatment resulted in both enhanced and altered anti-apoptotic dependency. Enhanced sensitivity to different BH3-mimetics after CHOP treatment was confirmed in specific cell lines, indicating heterogeneity of CHOP-induced resistance in DLBCL. Analysis of single CHOP compounds demonstrated that similar changes could also be induced by doxorubicin or vincristine, providing evidence for clinical combination therapies of doxorubicin or vincristine with BH3-mimetics in DLBCL. In conclusion, we show for the first time that CHOP treatment induces increased anti-apoptotic dependency on MCL-1 and BCL-XL, and not just BCL-2. These results provide new perspectives for the treatment of CHOP-resistant DLBCL and underline the potential of BH3 profiling in predicting therapy outcomes.

## 1. Introduction

The B-cell lymphoma 2 (BCL-2) family of proteins is a group of pro- and anti-apoptotic proteins with key roles in the regulation of intrinsic apoptosis. Proteins of the BCL-2 family share a general structure of one or more BCL-2 homology (BH) domains, of which expression of the BH3 domain is a universally shared feature. Expression of this BH3 domain is essential for protein-protein interactions, thereby allowing dimerization of pro-apoptotic sensitizer proteins (e.g., BAD, NOXA and HRK) with anti-apoptotic proteins (e.g., BLC-2, BCL-XL, MCL-1 and BCL-W). This interaction leaves the pro-apoptotic activator proteins (e.g., BID and BIM) free to interact with the effector proteins (e.g., BAX and BAK) to induce pore formation and cytochrome c release from the mitochondria, thus promoting apoptosis [1]. Dysregulated expression of several important anti-apoptotic proteins, such as BCL-2, plays a crucial role in carcinogenesis and anti-cancer therapy resistance [2]. The specific anti-apoptotic dependency in a particular cancer cell is often unknown, and cancer cells may adapt their anti-apoptotic dependencies when exposed to particular apoptosis-inducing therapies (e.g., chemotherapy) to further facilitate therapy resistance [3].

In diffuse large B-cell lymphoma (DLBCL), overexpression of the anti-apoptotic protein BCL-2 is correlated with adverse survival in patients treated with standard rituximab, cyclophosphamide, vincristine, doxorubicin, and prednisone (R-CHOP) [4]. In DLBCL patients, BCL-2 is commonly overexpressed due to either a t(14;18) chromosomal translocation or to BCL-2 amplification or transcriptional upregulation. The t(14;18) translocation is more common, at 20%–30% of cases, in the germinal center (GCB) molecular subtype, whereas BCL-2 amplification is found in 8%–30% of the activated B-cell (ABC) subtype [5,6].

Inhibition of the anti-apoptotic activity of BCL-2 has been suggested as a promising strategy for treatment [7], however, a single-agent phase I study of the specific BCL-2 inhibitor venetoclax in relapsed or refractory DLBCL reported limited efficacy [8]. On the other hand, promising results, including an overall response of 87.5% and a complete response (CR) of 79.2%, were reported in a phase I trial of venetoclax combined with R-CHOP as first-line treatment in non-Hodgkin lymphomas, including DLBCL [9]. However, other studies have reported that apoptotic resistance in DLBCL can be acquired through proteins other than BCL-2 [10]. Analysis of primary DLBCL patient samples treated with BH3 mimetic drugs (BCL-2 inhibitor and MCL-1 inhibitor) showed that patients could be subdivided into BCL-2-sensitive or MCL-1-sensitive subgroups [10]. However, the effect of standard (prolonged) CHOP treatment itself on DLBCL-specific pro- and anti-apoptotic signaling is currently unknown. To this end, we studied the effect of CHOP therapy on anti-apoptotic dependency in DLBCL using functional dynamic BH3 profiling [11]. By employing functional dynamic BH3 profiling, we compared the profiles of eight DLBCL cell lines before and after CHOP treatment and thus determined the change in dependency on anti-apoptotic proteins. Our results showed that CHOP resistance is not exclusively mediated through BCL-2, but by multiple other anti-apoptotic proteins. These results highlight the heterogeneity of anti-apoptotic dependency in DLBCL, which likely depends on other anti-apoptotic proteins in addition to BCL-2. These results could have implications for clinical trials evaluating efficacy of BH3 mimetics.

## 2. Results

### 2.1. DLBCL Cells Are Dependent on BCL-2 or MCL-1, but Not BCL-XL

**Figure 1:**
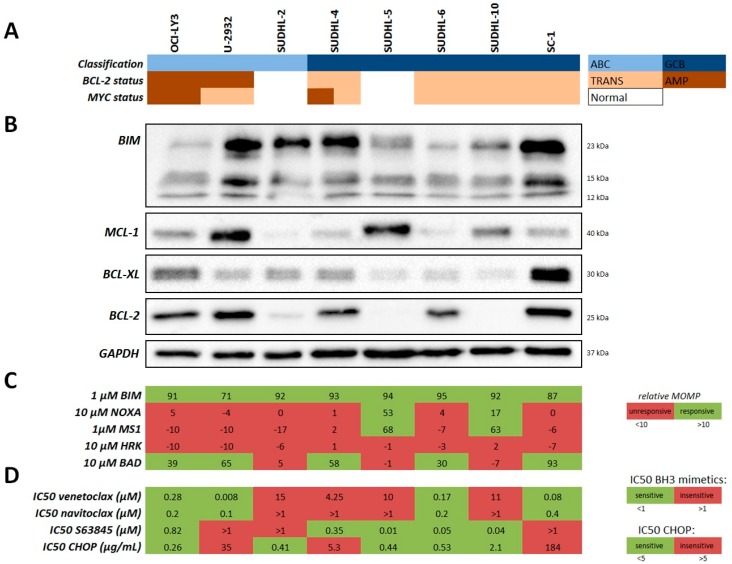
Expression and dependency on anti-apoptotic proteins in diffuse large B-cell lymphoma (DLBCL) cell lines. Overview of eight DLBCL cell lines with their (**A**) cell of origin (COO) classification, and translocation (TRANS) or amplification (AMP) status for B-cell lymphoma 2 (BCL-2) or MYC. (**B**) Protein expression of anti-apoptotic proteins encoded by BIM, MCL-1, BCL-XL and BCL-2. (**C**) BCL-2 homology (BH)3 profile peptide response as measured by mitochondrial outer membrane permeabilization (MOMP) (*n* = 3). Values marked in red were below 10% MOMP and therefore classified as unresponsive, whereas values marked in green were above 10% MOMP and classified as responsive. (**D**) The half maximal inhibitory concentration (IC_50_) values for venetoclax (BCL-2i; 48 h), navitoclax (BCL-2/XL/Wi; 48 h), S63845 (MCL-1i; 48 h) and cyclophosphamide, vincristine, doxorubicin, prednisolone (CHOP) chemotherapy (72 h) (*n* = 3). IC_50_ values below 1 µM (for venetoclax, navitoclax, or S63845) or below 5 µg/mL (for CHOP) were deemed sensitive and marked in red, whereas values above 1 µM (for venetoclax, navitoclax, or S63845) or above 5 µg/mL (for CHOP) were deemed insensitive and therefore marked in green.

BCL-2 protein overexpression is an important predictive and prognostic marker in DLBCL patients (4). We first validated the importance of different anti-apoptotic BCL-2 family protein expression in our representative DLBCL cell line panel with different genetic backgrounds (5 GCB and 3 ABC) and correlated anti-apoptotic protein expression to their functional anti-apoptotic dependency as determined by BH3 profiling (Figure 1, Figures S1 and S2).

All DLBCL cell lines showed protein expression of apoptotic activator BIM, indicating that cells are capable of undergoing apoptosis (Figure 1B). Variable protein expression of anti-apoptotic proteins MCL-1, BCL-XL, and BCL-2 was observed in the DLBCL cell lines, indicating that cells employed different and/or multiple anti-apoptotic proteins to protect from apoptosis. In most cases, cells with acquired translocation or amplification of the BCL-2 protein showed high expression of BCL-2, with the exception of cell line SUDHL-10, which showed high expression of MCL-1 instead (Figure 1A,B). Next, we performed BH3 profiling to determine the intrinsic functional dependency of cells on specific anti-apoptotic proteins (Figure 1C and Figure S1). BH3 profiling revealed that all cells showed a strong response to the BIM peptide (min 71%; max 95%), which confirms previous results for BIM protein expression (Figure 1B). In addition, cell lines with high BCL-2 protein expression showed a mitochondrial response to the BAD peptide (min 30%; max 93%), indicating functional dependency on BCL-2. Cell lines SUDHL-5 and SUDHL-10, which were not dependent on BCL-2, instead showed high response to the NOXA/MS1 peptides (min 17%; max 68%), indicating functional MCL-1 dependency, which matches with relatively high MCL-1 protein expression (Figure 1B). Together, these data demonstrate that DLBCL cells were either exclusively dependent on BCL-2 or MCL-1, but not on BCL-XL or multiple anti-apoptotic proteins simultaneously, despite expression of multiple anti-apoptotic proteins.

### 2.2. DLBCL Patients Show Simultaneous Expression of BCL-2, BCL-XL, and MCL-1

**Figure 2:**
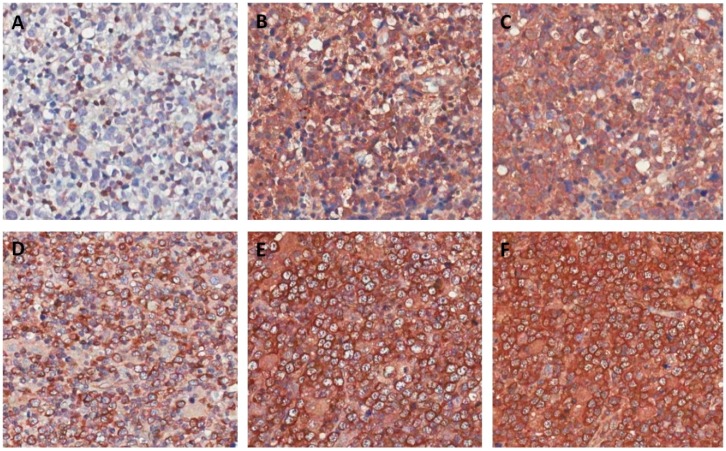
Immunohistochemistry staining for BCL-2, MCL-1, and BCL-XL in DLBCL. (**A**) Representative example of a BCL-2 negative DLBCL patient with positive staining for MCL-1 (**B**) and BCL-XL (**C**). (**D**) Representative example of a BCL-2 positive DLBCL patient with positive staining for MCL-1 (**E**) and BCL-XL (**F**). All images were captured at 200× magnification.

To validate that, like the DLBCL cell lines, DLBCL patients also show simultaneous expression of BCL-2, BCL-XL, and MCL-1, we performed immunohistochemistry staining on 55 DLBCL patient tissues (Table 1 and Figure 2).

Staining for the anti-apoptotic proteins revealed 34/55 patients (62%) stained positive for BCL-2, 55/55 (100%) stained positive for BCL-XL, and 52/55 (95%) stained positive for MCL-1. In addition, one case (2%) was positive for only one anti-apoptotic protein (BCL-XL), 22 cases (40%) were positive for two out of three anti-apoptotic proteins, and 32 cases (58%) were positive for all three anti-apoptotic proteins. Of the BCL-2 negative cases, 21/21 (100%) were positive for BCL-XL and 20/21 (95%) were positive for MCL-1 (Table 1 and Figure 2A–C). A similar trend was observed in the BCL-2 positive cases, of which 33/34 (97%) stained positive for BCL-XL and 32/34 (94%) were positive for MCL-1 (Table 1 and Figure 2D–F). Together, these data demonstrate that there are high levels and simultaneous expression of the multiple anti-apoptotic proteins, which strongly mirror our findings in the DLBCL cell lines.

### 2.3. BCL-2 Expression Predicts Sensitivity to Venetoclax and CHOP

**Figure 3:**
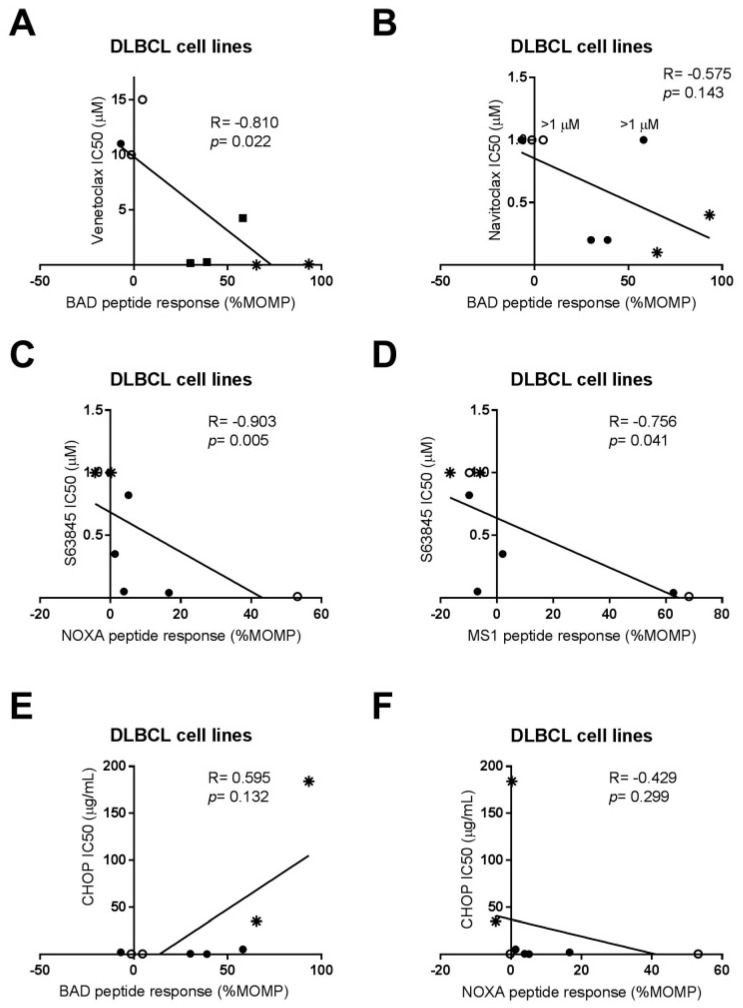
Correlation between BH3 response to BAD, NOXA, and MS1 peptides and sensitivity to anti-apoptotic inhibitors venetoclax (BCL-2i), navitoclax (BCL-2/XL/Wi), S63845 (MCL-1i), and CHOP chemotherapy in DLBCL cell lines. (**A**) Correlation between venetoclax IC_50_ versus BAD peptide response (%MOMP). (**B**) Correlation between navitoclax IC_50_ versus BAD peptide response (%MOMP). (**C**) Correlation between CHOP IC_50_ versus BAD peptide response (%MOMP). (**D**) Correlation between S63845 IC_50_ versus NOXA peptide response (%MOMP). (**E**) Correlation between S63845 IC_50_ versus MS1 peptide response (%MOMP). (**F**) Correlation between CHOP IC_50_ versus NOXA peptide response (%MOMP). Data were plotted as the mean of *n* = 3. Correlation was analyzed using Spearman’s correlation. Cell lines without a ‘double hit’ (MYC/BCL2) status are represented by open symbols (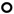) and cell lines with a ‘double hit’ status by closed symbols (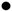). Cell lines with the highest BCL-2 protein expression (U-2932 and SC-1) are represented by an asterisk (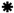).

Next, we correlated the intrinsic functional anti-apoptotic dependency of our cells to the sensitivity for anti-apoptotic inhibitors venetoclax (BCL-2i), S63845 (MCL-1i), navitoclax (BCL-2/BCL-XL/BCL-Wi), and CHOP chemotherapy (Figure 3).

We found a negative correlation between venetoclax IC_50_ and the BAD response (*r* = −0.810; *p* = 0.022) (Figure 3A), but not for navitoclax and BAD response (*r* = −0.575; *p* = 0.143) (Figure 3B), indicating that a high BAD response is predictive of a low venetoclax IC_50_ and thus high sensitivity to BCL-2 inhibition. This discrepancy between venetoclax and navitoclax is likely caused by the individual potency of the inhibitors, as venetoclax has a very high sensitivity for BCL-2 (Ki < 0.01 nM) compared to navitoclax (Ki ≤ 0.5 nM for BCL-XL and Ki ≤ 1 nM for BCL-2). On the other hand, cell lines with low or absent BCL-2 protein expression (SUDHL-2, SUDHL-5, and SUDHL-10) had a significantly higher IC_50_ for venetoclax (median IC_50_ 11 µM) compared to cell lines with high BCL-2 protein expression (OCI-LY3, U-2932, SUDHL-4, SUDHL-6, and SC-1; median IC_50_ for venetoclax of 0.17 µM; *p* = 0.0004) (Figure 1D and Figure S3A). In addition, functional BH3 profiling for NOXA (*r* = −0.903; *p* = 0.005) and MS1 (*r* = −0.756; *p* = 0.041) showed a strong correlation with sensitivity to S63845 (Figure 3C,D), whereas no correlation could be established between the S63845 sensitivity and MCL-1 protein expression (Figure S3C). These results demonstrate that, for venetoclax, both BCL-2 protein expression and functional BH3 profiling can predict sensitivity, whereas only functional BH3 profiling can predict sensitivity to S63845. Following these results, we correlated anti-apoptotic protein expression and the functional BH3 profiles to CHOP chemotherapy sensitivity and found that absence of BCL-2 protein expression was associated with low CHOP IC_50_ (median IC_50_ 0.44 µg/mL) (Figure 1D and Figure S3D), while high BCL-2 protein expression was associated with high CHOP IC_50_ values (median 5 µg/mL). These results are similar to findings in DLBCL patients, in which BCL-2 overexpression (with or without MYC rearrangement) are associated with poor survival in patients treated with R-CHOP (4). This correlation trend was also observed for the functional BCL-2 BH3 profile and CHOP response, in which a high BAD response showed a trend towards high CHOP IC_50_ values (*r* = 0.595; *p* = 0.1323) (Figure 3E). No correlation was observed with CHOP sensitivity and MCL-1 protein expression (Figure S3E) or the functional NOXA BH3 profile (Figure 3F). Taken together, these results show that in untreated/treatment naïve cells, both high BCL-2 expression and functional BH3 profiling for BAD can predict sensitivity to venetoclax and CHOP. However, in the situation that cells show no expression of BCL-2, functional BH3 profiling should be applied to determine sensitivity to specific anti-apoptotic inhibitors.

### 2.4. CHOP Chemotherapy Alters Dependency on Anti-Apoptotic Proteins

Based on previous experiments, we have established that DLBCL cells can express multiple anti-apoptotic proteins simultaneously, but usually show functional dependency on either BCL-2 or MCL-1. However, it remains unclear if standard CHOP treatment has the ability to alter or shift this dependency on anti-apoptotic proteins. To test this hypothesis, we employed dynamic BH3 profiling, which compares the BH3 profiles of treated and untreated cells and plots the differences in response to each BH3 peptide, revealing the altered anti-apoptotic dependencies after specific treatments. Dynamic BH3 profiling of CHOP-treated DLBCL cell lines revealed an enhanced mitochondrial response to the BIM peptide (OCI-LY3, SUDHL-5, SUDHL-6, SUDHL-10, and SC-1), indicating that cells had become more primed for apoptosis (Figure S4).

**Figure 4:**
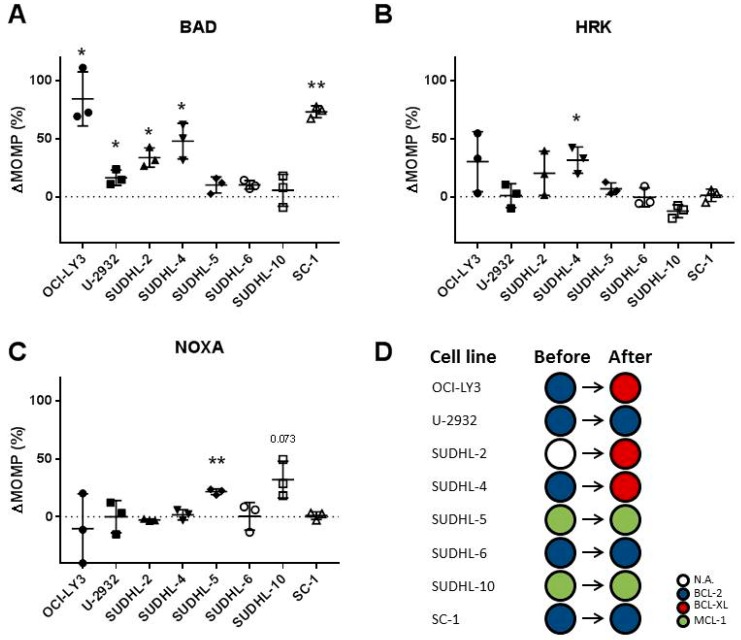
Dynamic BH3 profiles of DLBCL cell lines treated with CHOP chemotherapy. (**A**) Dynamic BH3 profiles for the DLBCL cell lines OCI-LY3 (1 µg/mL), U-2932 (10 µg/mL), SUDHL-2 (1 µg/mL), SUDHL-4 (10 µg/mL), SUDHL-5 (0.1 µg/mL), SUDHL-6 (1 µg/mL), SUDHL-10 (10 µg/mL), and SC-1 (10 µg/mL) after treatment with CHOP for 18 h for BAD, HRK (**B**) and NOXA (**C**). Delta mitochondrial outer membrane permeabilization (ΔMOMP%) was calculated by subtracting the percentage treated MOMP from percentage untreated MOMP. Data were plotted as the mean ± SD (*n* = 3). Statistical analysis was performed with a one-sample T-test (* *p* ≤ 0.05) (** *p* ≤ 0.01). (**D**) Schematic overview of the anti-apoptotic dependency in DLBCL cell lines before and after CHOP treatment. White = no specific dependency (N.A.), blue = BLC-2 dependency, red = BCL-XL dependency, and green = MCL-1 dependency.

In addition, five of the eight cell lines (OCI-LY3, U-2932, SUDHL-2, SUDHL-4, SUDHL-6, and SC-1) showed a mitochondrial response to the BAD peptide (Figure 4A), which could indicate that cells had become more dependent on BCL-2 after CHOP treatment. Apart from responses to BAD, three cell lines showed a strong response to the HRK peptide (SUDHL-2, OCI-LY3, and SUDHL-4) (Figure 4B), which could indicate enhanced dependency on BCL-XL, and two cell lines (SUDHL-5 and SUDHL-10) showed a response to the NOXA peptide (Figure 4C), which could indicate enhanced dependency on MCL-1 after CHOP treatment. Combined, these data suggest that CHOP treatment altered the BCL-2 dependency of cell lines OCI-LY3 and SUDHL-4 towards BCL-XL dependency, and made the anti-apoptotic independent cell line SUDHL-2 dependent on BCL-XL (Figure 4D). In the other cell lines, the cell specific anti-apoptotic dependency remained the same, however, it was further enhanced. None of these CHOP-induced changes were related to a specific cell of origin (COO) or BCL2/MYC status, suggesting that changes are COO and BCL-2/MYC independent. Taken together, these data demonstrate that CHOP chemotherapy not only enhances the anti-apoptotic dependency, it can also alter the anti-apoptotic dependency, demonstrating that upfront expression levels of anti-apoptotic proteins are insufficient to predict successful combination with anti-apoptotic inhibitors.

### 2.5. CHOP Chemotherapy Enhances Sensitivity to Anti-Apoptotic Inhibitors

To validate that CHOP treatment enhances or alters the cell specific dependency on anti-apoptotic proteins, we tested the combination of CHOP chemotherapy with venetoclax (BCL-2i), navitoclax (BCL-2/BCL-XL/BCL-Wi), or S63845 (MCL-1i) in the cell lines SC-1 (enhanced BCL-2 dependency), SUDHL-4 (altered BCL-2 → BCL-XL dependency), and SUDHL-10 (enhanced MCL-1 dependency).

**Figure 5:**
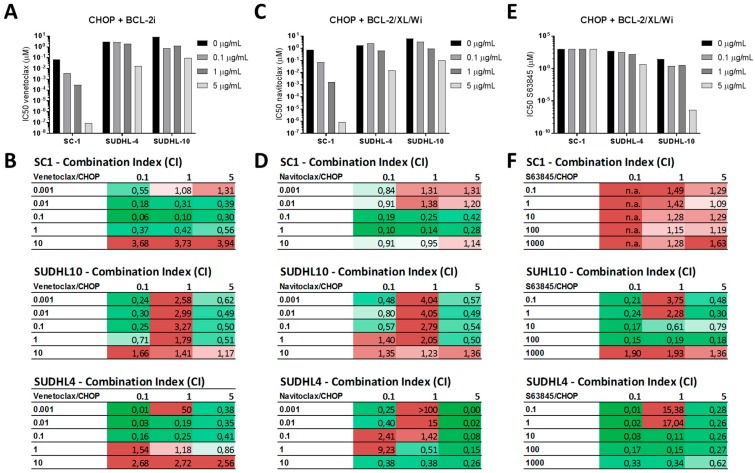
Effect of CHOP and BH3 mimetic combination treatments on viability in DLBCL cell lines. (**A**) IC_50_ values for venetoclax and CHOP combination therapy calculated from metabolic activity data in the cell lines SC-1, SUDHL-4, and SUDHL-10. Cells were treated for 72 h and data were normalized to control. Data were plotted as the mean ± SD (*n* = 3). (**B**) Synergism was determined by calculating the combination index (CI) from metabolic activity data for the venetoclax and CHOP combination therapy. Synergism was calculated with the Chou–Talalay method using CompuSyn. Synergistic combinations are depicted in green (CI < 1.0), additive combinations are depicted in white (CI = 1.0), and antagonistic combinations are depicted in red (CI > 1.0). (**C**) IC_50_ values for navitoclax and CHOP combination therapy calculated from metabolic activity data in SC-1, SUDHL-4, and SUDHL-10. Cells were treated for 72 h and data were normalized to control. Data were plotted as the mean ± SD (*n* = 3). (**D**) Synergism calculations from metabolic activity data for the navitoclax and CHOP combination therapy (*n* = 3). (**E**) IC_50_ values for S63845 and CHOP combination therapy calculated from metabolic activity data in cell lines SC-1, SUDHL-4, and SUDHL-10. Cells were treated for 72 h and data were normalized to control. Data were plotted as the mean ± SD (*n* = 3). (**F**) Synergism calculations from metabolic activity data for the S63845 and CHOP combination therapy (*n* = 3).

Treatment with CHOP induced a dose-dependent enhanced sensitivity to venetoclax in cell lines SC-1 and SUDHL-4 (Figure S5), resulting in a decrease in IC_50_ values for venetoclax (Figure 5A) and overall strong synergism for the combination of CHOP with venetoclax (Figure 5B). A similar effect was observed when CHOP was combined with navitoclax, which also induced a decrease in IC_50_ values (Figure 5C) and overall strong synergism (Figure 5D) in cell lines SC-1 and SUDHL-4. These changes were not observed in cell line SUDHL-10, as predicted by dynamic BH3 profiling. Instead, SUDHL-10 showed a strong decrease in IC_50_ values for S63845 (Figure 5E) and overall strong synergism for the combination of CHOP with S63845 (Figure 5F). Together, these data demonstrate that CHOP chemotherapy can significantly enhance the effectivity of anti-apoptotic inhibitors in DLBCL.

**Figure 6:**
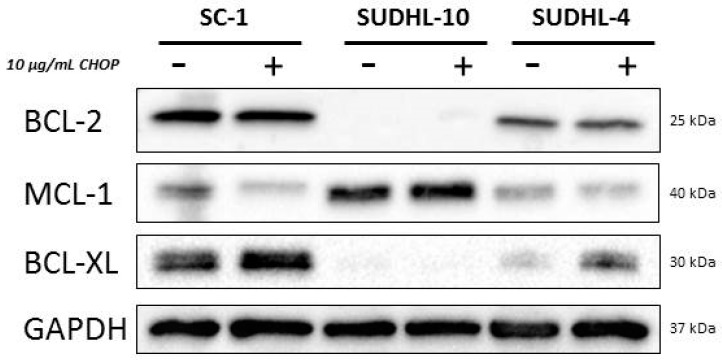
Western blot of anti-apoptotic proteins after CHOP treatment in DLBCL cell lines. Western blot of anti-apoptotic proteins BCL-2, MCL-1, BCL-XL, and loading control GAPDH in the DLBCL cell lines SC-1, SUDHL-10 and SUDHL-4 treated with 10 µg/mL CHOP for 24 h.

In addition to testing the synergy for CHOP together with anti-apoptotic inhibitors, we also investigated whether CHOP treatment would alter the expression levels of anti-apoptotic proteins BCL-2, MCL-1, or BCL-XL (Figure 6).

Despite the increased sensitivity to venetoclax and the changes observed in the dynamic BH3 profiles for the cell lines SC-1 and SUDHL-4, no changes were observed in BCL-2 protein levels after CHOP treatment. Similarly, no changes were observed in MCL-1 protein expression after CHOP treatment in SUDHL-10, despite the strong dynamic BH3 response and enhanced sensitivity for MCL-1 inhibitor S63845. However, protein expression of BCL-XL was increased after CHOP treatment in both SC-1 and SUDHL-4, but not in SUDHL-10. These findings are in line with the observed HRK response in the BH3 profile found in SUDHL-4, although an increased HRK response was not observed in SC-1. These results suggest that while CHOP treatment can change expression levels of anti-apoptotic proteins, these changes may not accurately reflect the dependency of cells on these anti-apoptotic proteins.

In conclusion, we were able to validate that CHOP treatment enhances the dependency on anti-apoptotic proteins, without necessarily altering the expression levels of the anti-apoptotic proteins. Importantly, high levels of synergism could be achieved for CHOP with anti-apoptotic inhibitors. Therefore, performing functional assays such as dynamic BH3 profiling is essential to establish how anti-apoptotic dependency may have changed as a result of chemotherapeutic pressure.

### 2.6. Vincristine and Doxorubicin Change Dependency on Anti-Apoptotic Proteins

**Figure 7:**
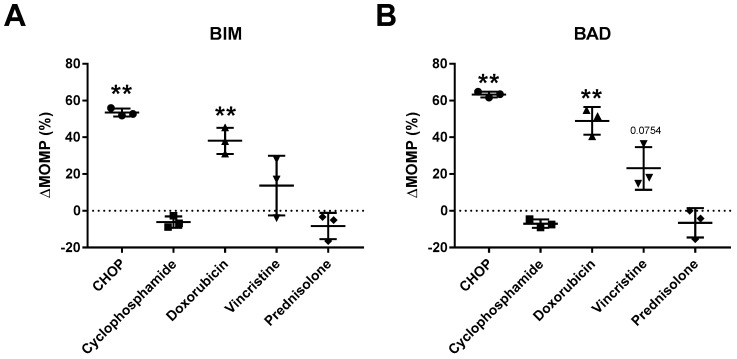
Dynamic BH3 profiles of SC-1 treated with individual CHOP compounds. Dynamic BH3 profile of (**A**) BIM and (**B**) BAD in the SC-1 cell line treated for 18 h with 10 µg/mL CHOP, 31.7 µM cyclophosphamide, 18 nM vincristine, 1 µM doxorubicin, or 3.18 µM prednisolone. Delta mitochondrial outer membrane permeabilization (ΔMOMP%) was calculated by subtracting the percentage treated MOMP from the percentage untreated MOMP. Data were plotted as the mean ± SD (*n* = 3). Statistical analysis was performed using a one-sample T-test (** *p* ≤ 0.01).

In previous experiments we demonstrated that CHOP chemotherapy can greatly alter or enhance the dependency on anti-apoptotic proteins, resulting in synergism with anti-apoptotic inhibitors. However, since CHOP chemotherapy is composed of cyclophosphamide (C), doxorubicin (H), vincristine (O) or prednisolone (P), it remains unclear which of these compounds is responsible for the induced effects. We therefore tested each individual compound at the representative dosage of 10 µg/mL CHOP in a clinical ratio of 83/5.5/0.16/11.1, respectively [12] in the representative cell line SC-1 (Figure 7).

Dynamic BH3 profiling of cells treated with vincristine or doxorubicin showed a similar response to the BIM (Figure 7A) and BAD (Figure 7B) peptide when compared to the full CHOP regimen, albeit somewhat lower. The combined effects for vincristine and doxorubicin on BIM (BIM Δ38% + Δ4% = Δ52%) were similar compared to the effects induced by CHOP (BIM Δ54%), which was also observed for BAD (BAD Δ49% + Δ23% = Δ72% vs. Δ63% by CHOP). No changes were induced by cyclophosphamide or prednisolone. So, although the greatest effect on anti-apoptotic dependency is achieved with complete CHOP, the bulk of the effect can likely be attributed to the actions of vincristine and doxorubicin, indicating that vincristine and doxorubicin are suitable chemotherapeutics in combination with BH3 mimetic drugs.

## 3. Discussion

While the relationship between BCL-2 expression and CHOP therapy resistance is well established [13,14], it has remained unclear how CHOP treatment affects anti-apoptotic proteins in DLBCL. Using functional BH3 profiling, we showed for the first time that CHOP treatment enhances dependency on anti-apoptotic proteins, making cells more sensitive to BH3 mimetic drugs, most likely through vincristine and/or doxorubicin. We also confirmed that CHOP treatment increases the sensitivity of DLBCL cells to BH3 mimetics, highlighting the potential for clinical applications. Furthermore, our findings highlighted the heterogeneity of anti-apoptotic dependency in DLBCL, in that CHOP treatment resulted in enhanced dependency, not only on BCL-2, but also on MCL-1 and BCL-XL. Since we found similar heterogeneity of anti-apoptotic expression in DLBCL patients, it seems plausible that in patients a similar enhanced or altered pattern on anti-apoptotic proteins might occur. A recent study by Smith et al. confirms this heterogeneity in DLBCL, as they demonstrated in a similar fashion how DLBCL cell lines show specific dependency on either BCL-2, BCL-XL, or MCL-1 for survival [15]. None of the observed changes were related to a specific COO in our DLBCL cell line panel, indicating that initial inhibitor sensitivity or CHOP-induced anti-apoptotic changes might not be related to the differentiation stadium at which DLBCL might occur. In addition, we observed anti-apoptotic changes after CHOP treatment in both ‘double-hit’ (double *MYC/BCL-2* translocation) and non-hit cell lines. It has been recently suggested that BCL-2, but not MCL-1, inhibition is effective in double-hit DLBCL [16]. However, as we have shown that double-hit status does not always match protein expression (e.g., SUDHL-10 lacks BCL-2 expression), this could result in ineffective treatment. In fact, we saw an effective response to MCL-1 inhibitor treatment in the BCL-2/MYC double-hit SUDHL-10 cell line, both prior to CHOP treatment and after CHOP treatment, whereas no response was observed to BCL-2 inhibition. Although our data are broadly in line with recently published clinical trial data for venetoclax in NHL [8], we found a potential impact for MCL-1 dependency after CHOP treatment, which might indicate that a significant number of patients would derive greater benefit from a MCL-1 inhibitor than from venetoclax treatment.

Robust immunohistological chemical staining for BCL-2 expression and the prognosis of R-CHOP treated patients are both well established. However, as this is not the case for MCL-1 and other BCL-2 family members, it is still impossible to predict the prognosis of R-CHOP-treated patients based on the expression of anti-apoptotic proteins. Alternatively, dynamic BH3 profiling would be a very useful tool to predict an anti-apoptotic response to CHOP chemotherapy when applied to treatment-naïve fresh DLBCL patient samples. However, in routine clinical practice, dynamic BH3 profiling of DLBCL samples is challenging, as tissue samples are scarce and dividing cells are required in order to observe possible effects of chemotherapy (e.g., CHOP) on the subsequent BH3 response in vitro. Ex vivo culture of DLBCL is not possible without stimulation, and stimulation of cells (e.g., CD40 ligand) might skew and alter the BH3 response to chemotherapy. Further research and optimization of BH3 profiling is therefore warranted. In addition, in vivo experiments with either a patient-derived xenograft (PDX) model or a spontaneous DLBCL mouse model should preferably be performed to predict and validate the clinical applications of combining CHOP chemotherapy with a broad range of BH3 mimetic drugs.

Besides identifying MCL-1 and BCL-XL as novel contributors to CHOP-induced resistance, our data also show the therapeutic potential of vincristine and doxorubicin in combination with BH3 mimetics. In the current setting, the 10 µg/mL CHOP treatment contained single compound concentrations of 31.7 µM cyclophosphamide, 18 nM vincristine, 1 µM doxorubicin, and 3.18 µM prednisolone, which are all within the range of clinical maximal plasma levels in patients (128 µM cyclophosphamide, 7 nM vincristine, 6.73 µM doxorubicin, and 0.145 µM prednisolone) [17]. At the moment, clinical trials are underway regarding liposomal vincristine and venetoclax in relapsed or refractory T-cell or B-cell NHL (NCT03504644/clinicaltrials.gov). Based on our data, we predict that both vincristine and doxorubicin will induce changes in anti-apoptotic dependency, although whether changes will always favor BCL-2 rather than MCL-1 or BCL-XL will remain unclear without functional dBH3 profiling. In addition, additional experiments with higher concentrations of cyclophosphamide might induce similar anti-apoptotic changes, which warrants further investigation for cyclophosphamide with BH3 mimetic drugs.

In conclusion, we demonstrated the potential of BH3 profiling as a predictor of anti-apoptotic dependencies in DLBCL. Moreover, we showed that CHOP treatment induces increased anti-apoptotic dependency, not only on BCL-2, but also on MCL-1 and BCL-XL. As a result, patient-specific BH3 mimetic treatment might well lead to synergistic lethality and reduce unnecessary treatment. Although BCL-2 remains one of the primary actors in anti-apoptotic resistance, our results indicate that caution is advisable and that other BH3 mimetics should not be neglected as they might well influence or alter therapy outcomes.

## 4. Materials and Methods

### 4.1. Cell Lines and Culture Conditions

The DLBCL cell lines U-2932, SUDHL-2, SUDHL-4, and SC-1 were cultured in suspension in Roswell Park Memorial Institute medium 1640 (RPMI 1640; Lonza BioWhittaker, Walkersville, MD, USA) with 10% fetal bovine serum (FBS; HyClone Thermo Scientific, Waltham, MA, USA), 1% penicillin-streptomycin (PS; Lonza BioWhittaker) and 1% glutamine (Lonza BioWhittaker). The DLBCL cell lines OCILY3, SUDHL-5, SUDHL-6, and SUDHL-10 were cultured in suspension in RPMI 1640 with 20% FBS, 1% PS, and 1% glutamine. All cell lines were cultured at 37 °C with 5% CO2 in a humidified atmosphere. Cell lines were obtained from ATCC and DSMZ. The identity of our cell lines was checked on a regular basis. Cell of origin (COO), BCL-2, and MYC status was based on data from ATCC and DSMZ.

### 4.2. Patient Material

Patient material was used from 55 DLBCL patients with primary DLBCL, including most subtypes. Material was acquired in accordance with international regulations and professional guidelines (the Declaration of Helsinki and the International Conference on Harmonization Guidelines for Good Clinical Practice). Material used in this project (RR#201800551, 1 November 2018) was obtained from anonymous rest material. The medical ethics review board (Central Ethics Review Board non-WMO studies, University Medical Center Groningen (UMCG)) waives the need for approval if rest material is used, under the law in the Netherlands and waives the need for informed consent when patient anonymity is assured.

### 4.3. Immunohistochemistry (IHC)

IHC was performed with antibodies against BCL-2 (M0887, Dako, Glostrup, Denmark), BCL-XL (SC7195, Santa Cruz Biotechnology, Santa Cruz, CA, USA) and MCL-1(A3534, Dako, Glostrup, Denmark) on paraffin-embedded tissue sections after antigen retrieval (pH 6, pH 9, and pH 6, respectively). Staining was visualized using HRP-labeled secondary antibodies (Dako) and 3,3′-diaminobenzidine (Sigma–Aldrich, St Louis, MO, USA). Appropriate positive and negative controls were performed for each staining. The cases were stained and scored on a tissue micro-array (TMA). In the TMA, each case was represented by three tissue cores.

### 4.4. Compounds

The BCL-2 inhibitor venetoclax (#S8048, Houston, TX, USA), the BCL-2/BCL-XL/BCL-W inhibitor navitoclax (#S1001), and the MCL-1 inhibitor S63845 (#S8383) were all acquired from Selleckchem. CHOP chemotherapy consisted of cyclophosphamide (University Medical Center Groningen (UMCG) pharmacy, Groningen, The Netherlands), doxorubicin (#S1208, Selleckchem, Houston, TX, USA), vincristine (UMCG pharmacy, Groningen, the Netherlands), and prednisolone (#S1737, Selleckchem), in a composition set at the clinical ratio of 83/5.5/0.16/11.1, respectively (12).

### 4.5. BH3 Profiling-Plate-Based Assay

BH3 profiling is a method to functionally characterize the interplay between anti- and pro-apoptotic proteins for a particular cell line by employing a panel of pro-apoptotic BH3 domain peptides to measure specific anti-apoptotic protein reactions. Upon treatment with these peptides, the mitochondrial outer membrane response can be measured by cytochrome c release from the mitochondria using flow cytometry or as the altered mitochondrial membrane potential by measuring fluorescence emission of the JC-1 dye in a plate-based assay [3]. The BIM peptide is a pro-apoptotic activator protein that reacts with multiple anti-apoptotic BH-3 proteins and can be used to establish whether cells are able to undergo intrinsic apoptosis [11]. To determine the specific anti-apoptotic dependency of a DLBCL cell line, we looked at the mitochondrial response of cells to the pro-apoptotic sensitizers BAD, HRK, NOXA, or MS1. Cells dependent on anti-apoptotic BCL-2 will release cytochrome c in response to the BAD peptide, which interacts with BCL-2, BCL-XL, and BCL-W, but not HRK (which interacts only with BCL-XL). If cells are dependent on BCL-XL, cytochrome c release will be observed in response to both BAD and HRK peptides. Cells dependent on anti-apoptotic MCL-1 will show a response to NOXA and MS1.

In order to establish the effect of CHOP with BH3, profiling cells were incubated at a density of 1.0 × 10^6^ cells/mL for 18 h with CHOP. After incubation, cells were washed in mannitol experimental buffer (150 mM D-mannitol (M9647 Sigma–Aldrich, 3050 Spruce St., St. Louis, MO, USA), 10 mM HEPES (H3375 Sigma–Aldrich), 50 mM KCl (1.04936 Merck KGaA, Darmstadt, Germany), 20 nM EGTA (E4378 Sigma–Aldirch), 20 nM EDTA (11280, Serva Electrophoresis GmbH, Carl-Benz-Str. 7, Heidelberg, Germany), 0,1% bovine serum albumin (BSA) (11930 Serva Electrophoresis GmbH), 5 mM succinate (1.00682 Merck KGaA) dissolved in dH_2_O, pH 7.5 (MEB)) and resuspended at 3.2 × 10^6^ cells/mL in MEB. The cell suspension was mixed 1:1 with 4 µM JC-1 permeabilization/staining solution (ENZ-52304, Enzo Life Sciences, 10 Executive Blvd., Farmingdale, NY 11735, USA), 0.004% digitonin (1500 643, Boehringer Mannheim GmbH, Mannheim, Germany), 20 mM β-mercaptoethanol (8.05740 Merck KGaA), and 40 µg/mL oligomycin (O4876, Sigma–Aldrich) prepared in MEB) and incubated at room temperature for 10 min in the dark. BIM, PUMA, BAD, NOXA, HRK, BMF, and PUMA2A (JPT Peptide Technologies GmbH, Volmerstrasse, Berlin, Germany) were prepared in MEB in a black flat-bottom non-treated polystyrene 96-well plate (3915 Costar, Corning Incorporated, 2 Alfred Road, Kennebunk, ME, USA). Peptide sequences used for the assay were identical to those described by Ryan and Letai [18]. Plates were either used directly or sealed (Silverseal sealer, ref 676090, Greiner-Bio-One GmbH, Mayback Str., Frickenhausen, Germany), frozen at −80 °C and thawed for one hour at room temperature before use. Cells in permeabilization/staining solution were added to the plate 1:1 at a final volume of 100 µL and shaken for 15 s, followed by measurement of fluorescence (excitation 545 nm, emission 590 nm) every 5 min for 2 h at 30 °C (Varioskan). All experiments were performed in triplicate or quadruplicate. Area under the curve (AUC) was calculated as the percentage mitochondrial outer membrane permeabilization (MOMP) and normalized to PUMA2A (negative control) and FCCP (positive control) with the formula:

1 − ((AUC sample − AUC FCCP) ÷ (AUC PUMA2A − AUC FCCP)) × 100%
(1)

The dynamic BH3 profile (ΔMOMP) was calculated by subtracting the percentage treated MOMP from the percentage untreated MOMP. BH3 profiling with a mean ΔMOMP ≥ 20% were classified as biologically relevant, even if they were not always statically significant, as they often lead to significant enhanced sensitivity to BH3 mimetic drugs, indicating biological relevance.

### 4.6. Flow Cytometry—Cell Viability for IC_50_

For cell viability analysis, 0.2 × 10^6^ cells/mL were treated for the appropriate times, washed with 1% BSA/PBS and resuspended in propidium iodide solution (P4170, Sigma). Samples were processed on a BD FACSCalibur 2 and analyzed with ModFit LT (Verity Software House, version 4.1.7, Topsham, ME, USA). Experiments were performed three times.

### 4.7. Resazurin—Metabolic Viability for Combination Therapies

After treatment of 0.4 × 10^6^ cells/mL, 1/20th of the total volume of resazurin (AlamarBlue, Thermo Fisher Scientific, Waltham, MA, USA) was incubated for nine hours prior to read-out (Varioskan, excitation 560 nm, emission 590 nm). Experiments were performed 3 times.

### 4.8. Western Blot

Western blot was performed as previously described [19]. Primary antibodies used were anti-BIM (1:1000, #2933 (C34C5) Cell Signaling Technology, Danvers, MA, USA), anti-MCL-1 (1:1000, #5453 (D35A5) Cell Signaling Technology), anti-BCL-2 (1:1000, ab32124 (E17), Abcam, Cambridge, United Kingdom), and anti-BCL-XL (1:1000, #2764 (54H6) Cell Signaling Technology).

### 4.9. Statistical Analysis

Data were analyzed using Graphpad PRISM (version 5.0, GraphPad Software, San Diego, CA, USA) software and tested for significant differences with a paired T-test. Correlation was analyzed using Spearman’s correlation. Combination index (CI) was calculated using the Chou-Talalay method and CompuSyn software (ComboSyn Inc, NJ, USA). * indicates *p* ≤ 0.05, ** indicates *p* ≤ 0.01, and *** indicates *p* ≤ 0.001.

## Figures and Tables

**Table 1:** Immunohistochemistry of BCL-2, BCL-XL and MCL-1 in DLBCL patients (*n* = 55).

DLBCL Cases	BCL-2 Negative (*n* = 21)	BCL-2 Positive (*n* = 34)
BCL-XL negative	0/21 (0%)	1/34 (3%)
BCL-XL positive	21/21 (100%)	33/34 (97%)
MCL-1 negative	1/21 (5%)	2/34 (6%)
MCL-1 positive	20/21 (95%)	32/34 (94%)

## Supplementary Materials

Supplementary materials can be found at https://www.mdpi.com/1422-0067/20/23/6036/s1.
