# Supplementary material for: Heterogeneous Pattern of Dependence on Anti-Apoptotic BCL-2 Family Proteins upon CHOP Treatment in Diffuse Large B-Cell Lymphoma
Source: Int J Mol Sci. 2019 Nov 30;20(23):6036. doi: 10.3390/ijms20236036 (PMC6928684; doi:10.3390/ijms20236036)
Supplement: Supplementary file 1 [file ijms-20-06036-s001.zip › SupplementalFigure2.pdf]

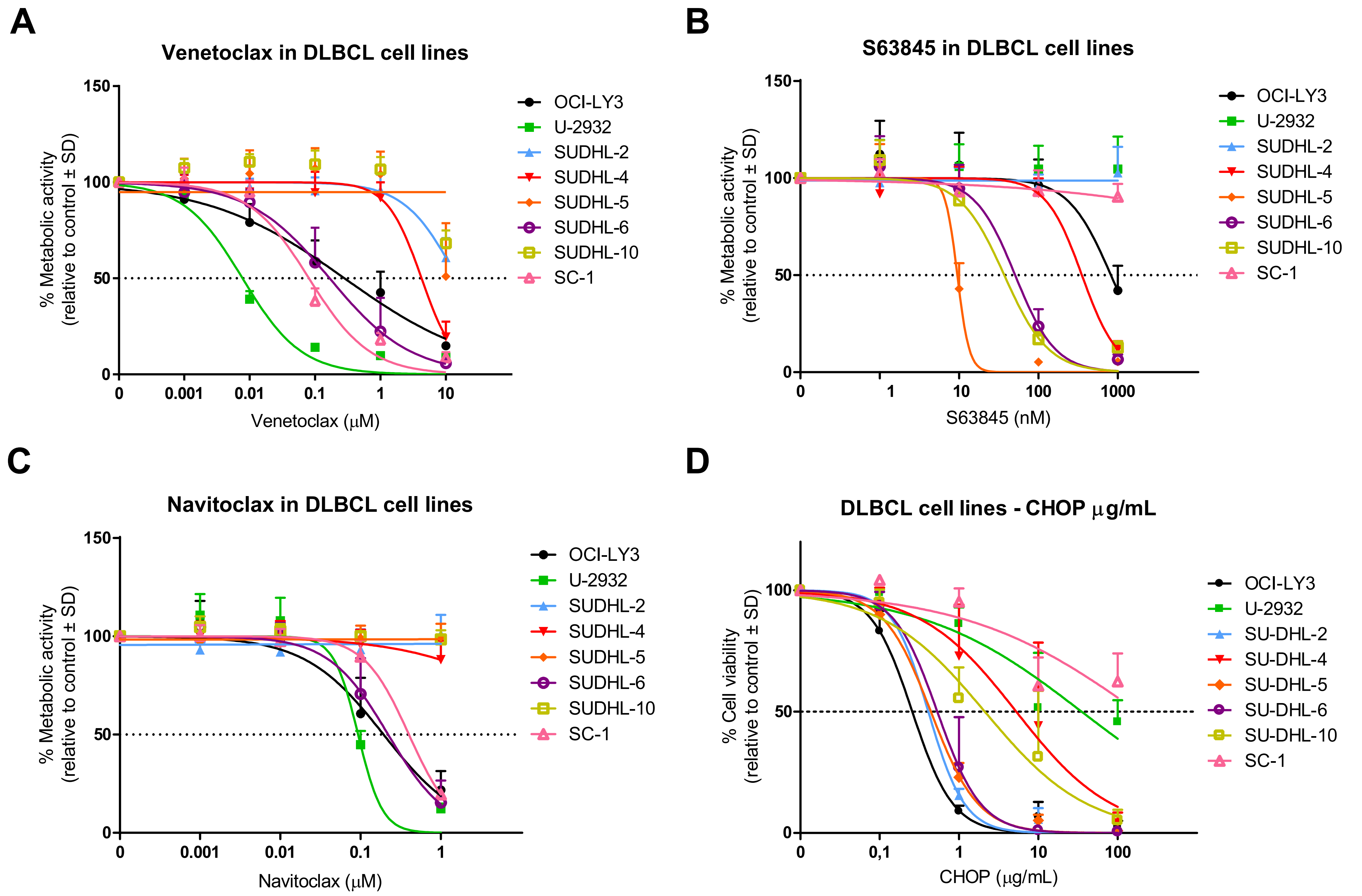

A
Venetoclax in DLBCL cell lines
% Metabolic activity (relative to control ± SD)
150
100
50
0
0
0.001
0.01
0.1
1
10
Venetoclax (μM)
OCI-LY3
U-2932
SUDHL-2
SUDHL-4
SUDHL-5
SUDHL-6
SUDHL-10
SC-1
B
S63845 in DLBCL cell lines
% Metabolic activity (relative to control ± SD)
150
100
50
0
0
1
10
100
1000
S63845 (nM)
OCI-LY3
U-2932
SUDHL-2
SUDHL-4
SUDHL-5
SUDHL-6
SUDHL-10
SC-1
C
Navitoclax in DLBCL cell lines
% Metabolic activity (relative to control ± SD)
150
100
50
0
0
0.001
0.01
0.1
1
Navitoclax (μM)
OCI-LY3
U-2932
SUDHL-2
SUDHL-4
SUDHL-5
SUDHL-6
SUDHL-10
SC-1
D
DLBCL cell lines - CHOP μg/mL
% Cell viability (relative to control ± SD)
100
50
0
0
0,1
1
10
100
CHOP (μg/mL)
OCI-LY3
U-2932
SU-DHL-2
SU-DHL-4
SU-DHL-5
SU-DHL-6
SU-DHL-10
SC-1
