# Supplementary material for: Heterogeneous Pattern of Dependence on Anti-Apoptotic BCL-2 Family Proteins upon CHOP Treatment in Diffuse Large B-Cell Lymphoma
Source: Int J Mol Sci. 2019 Nov 30;20(23):6036. doi: 10.3390/ijms20236036 (PMC6928684; doi:10.3390/ijms20236036)
Supplement: Supplementary file 1 [file ijms-20-06036-s001.zip › Supplemental_DLBCL_dependence_on_anti-apoptotic_IJoMS.docx]

**Supplemental data**


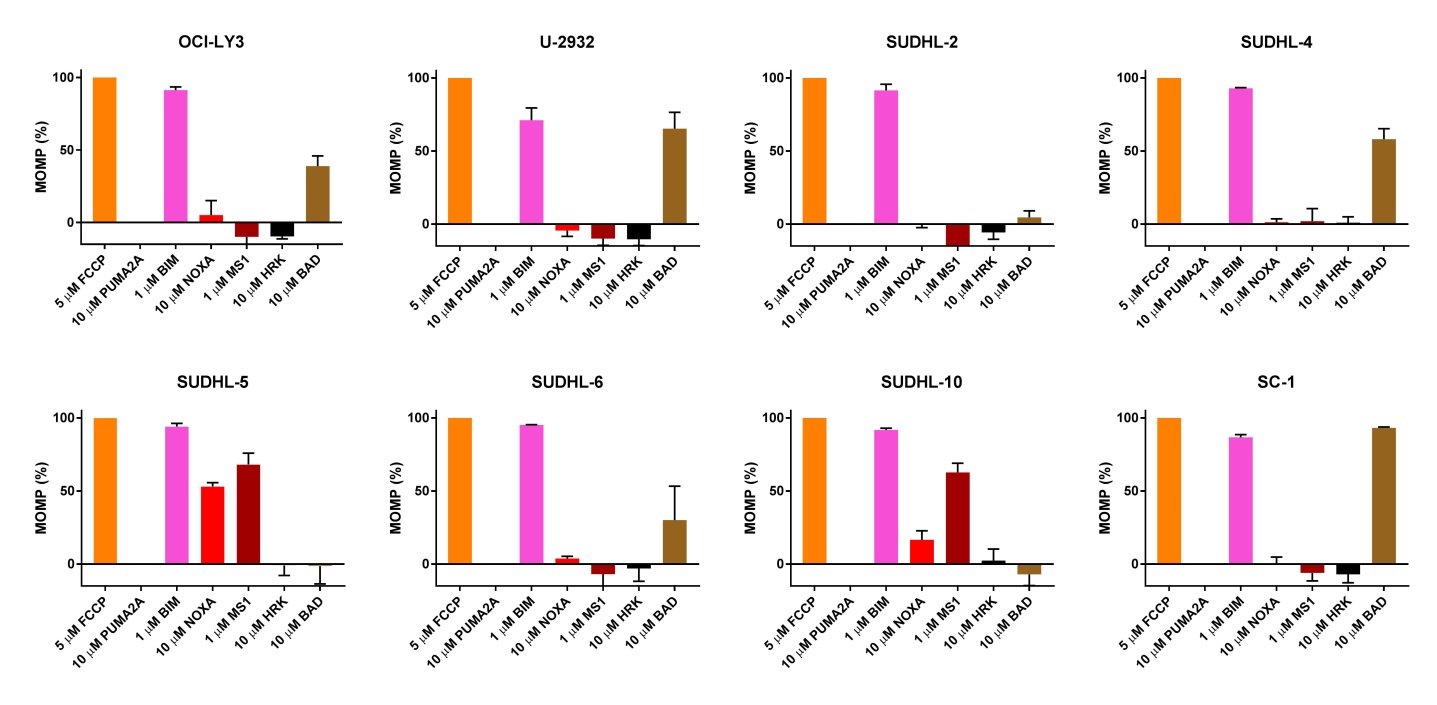


**Supplemental figure 1. Static BH3 profiles of DLBCL cell lines.**Static BH3 profiles of DLBCL cell lines, expressed as the percentage mitochondrial outer membrane permeabilization (MOMP%) upon treatment with BH3 peptides (*n*=3). FCCP and PUMA2A were used as positive and negative controls, respectively.

**
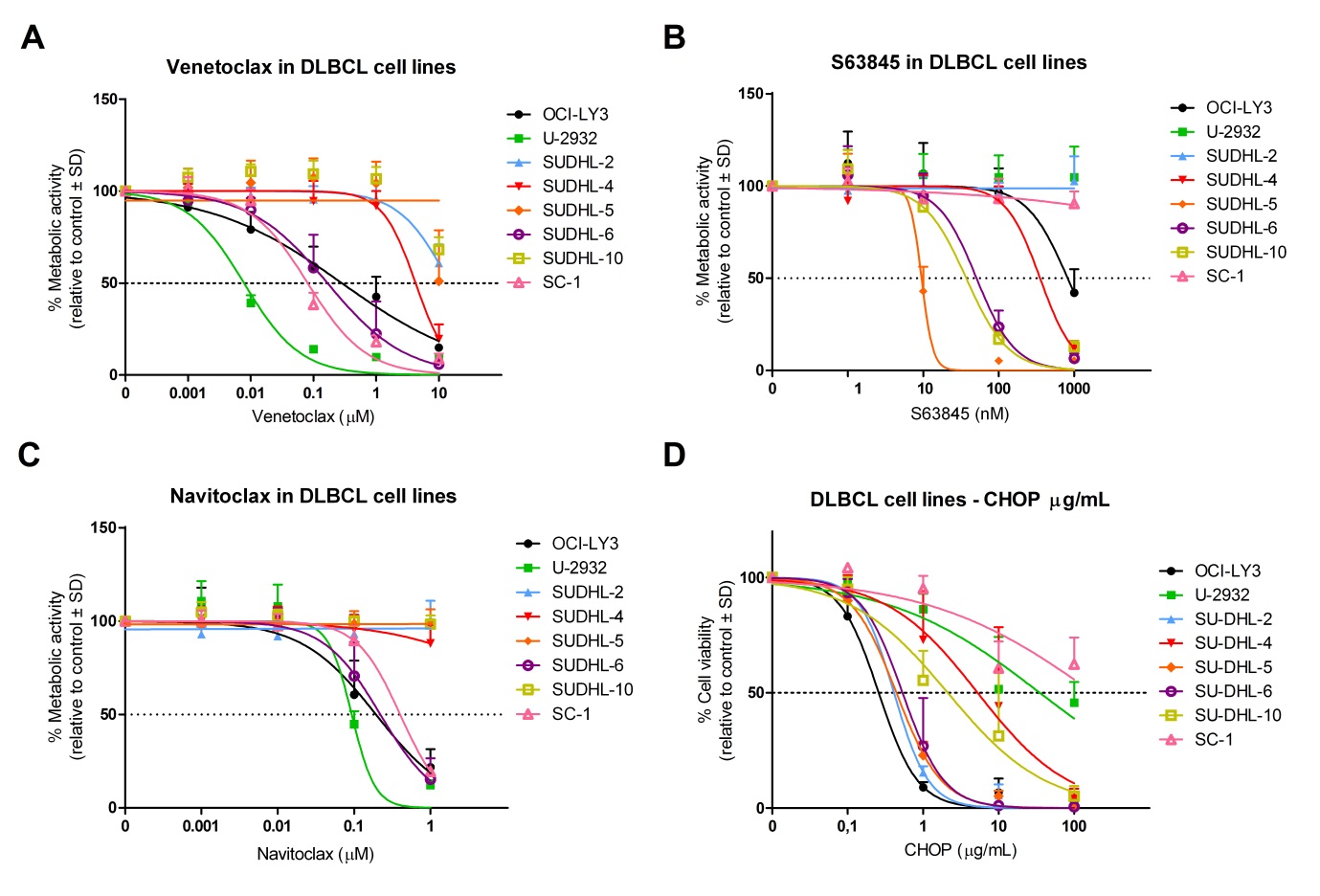

Supplemental figure 2. Dose-response curves of DLBCL cell lines treated with BH3 mimetics.**
A) Dose-response curve of eight DLBCL cell lines treated with the BCL-2 inhibitor venetoclax for 48 hours (*n*=3). B) Dose-response curve of eight DLBCL cell lines treated with the MCL-1 inhibitor S63845 for 48 hours (*n*=3). C) Dose-response curve of eight DLBCL cell lines treated with the BCL-2/BCL-XL/BCL-W inhibitor navitoclax for 48 hours (*n*=3). D) Dose-response curve of eight DLBCL cell lines treated with CHOP chemotherapy (cyclophosphamide, doxorubicin, vincristine and prednisolone) for 72 hours (*n*=3).

**Supplemental figure 3. Relation between BCL-2 and MCL-1 protein expression and sensitivity to BH3 mimetics and CHOP chemotherapy in DLBCL cell lines.**

A) Venetoclax IC_50_ plotted based on the absence or presence of BCL-2 protein expression for DLBCL cell lines. B) Navitoclax IC_50_ plotted based on the absence or presence of BCL-2 protein expression for DLBCL cell lines. C) S63845 IC_50_ plotted based on the absence or presence of MCl-1 protein expression for DLBCL cell lines. D) CHOP IC_50_ plotted based on the absence or presence of BCL-2 protein expression for DLBCL cell lines. E) CHOP IC_50_ plotted based on the absence or presence of MCl-1 protein expression for DLBCL cell lines. Statistical analysis was performed with two-tailed paired T-test (*p≤0.05) (**p≤0.01). Cell lines without a ‘double hit’ (MYC/BCL2) status are represented by open symbols (🞆) and cell lines with a ‘double hit’ status by closed symbols (⚫). Cell lines with the highest BCL-2 protein expression are represented by an asterisk (🞿).

**Supplemental figure 4. Dynamic BH3 profiles of DLBCL cell lines treated with CHOP chemotherapy.**A) Dynamic BH3 profiles for the DLBCL cell lines OCI-LY3 (1 µg/mL), U-2932 (10 µg/mL), SUDHL-2 (1 µg/mL), SUDHL-4 (10 µg/mL), SUDHL-5 (0.1 µg/mL), SUDHL-6 (1 µg/mL), SUDHL-10 (10 µg/mL) and SC-1 (10 µg/mL) after treatment with CHOP for 18 hours for BIM. Delta mitochondrial outer membrane permeabilization (ΔMOMP%) was calculated by subtracting the percentage treated MOMP from percentage untreated MOMP. Data were plotted as the mean ± SD (*n*=3). Statistical analysis was performed with a one-sample T-test (*p≤0.05) (**p≤0.01).

**
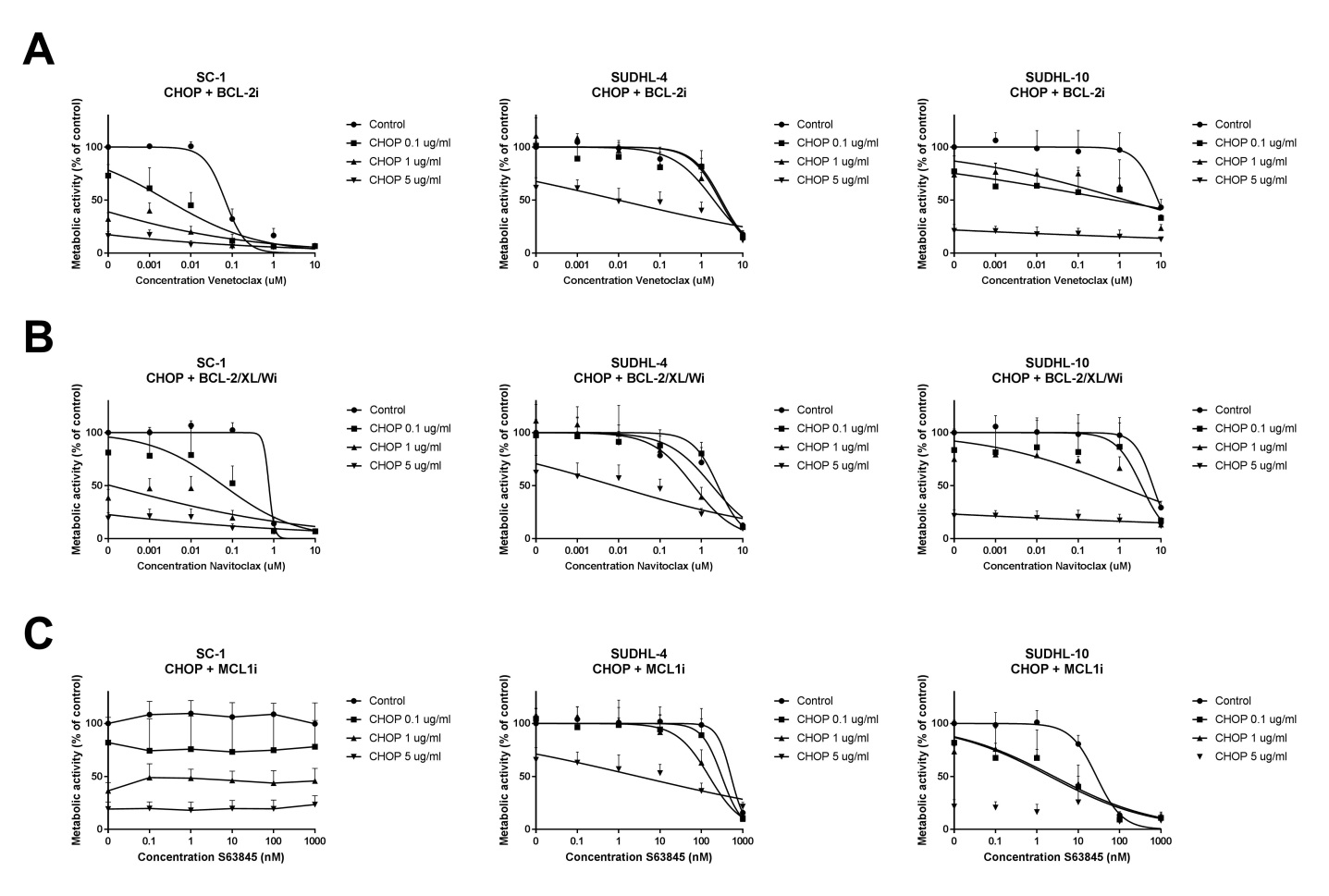
**

**Supplemental figure 5. Combined effect of CHOP and BH3 mimetics.**A) Metabolic activity of the DLBCL cell lines SC-1, SUDHL-4 and SUDHL-10 treated with CHOP and venetoclax (BCL-2i) for 72 hours, measured with resazurin. B) Metabolic activity of the SC-1, SUDHL-4 and SUDHL-10 cell lines treated with CHOP and navitoclax (BCL-2/XL/Wi) for 72 hours, measured with resazurin. C) Metabolic activity of SC-1, SUDHL-4 and SUDHL-10 treated with CHOP and S63845 (MCL-1i) for 72 hours, measured with resazurin. Data were normalized to the control and plotted as the mean ± SD (*n*=3).
