# Supplementary figures and images for: Heterogeneous Pattern of Dependence on Anti-Apoptotic BCL-2 Family Proteins upon CHOP Treatment in Diffuse Large B-Cell Lymphoma
Source: Int J Mol Sci. 2019 Nov 30;20(23):6036. doi: 10.3390/ijms20236036 (PMC6928684; doi:10.3390/ijms20236036)

**A**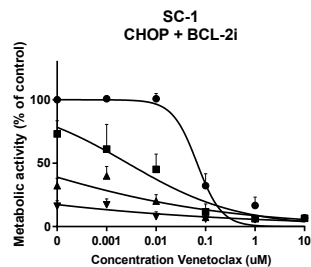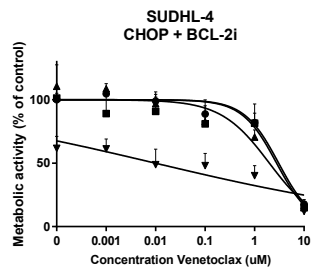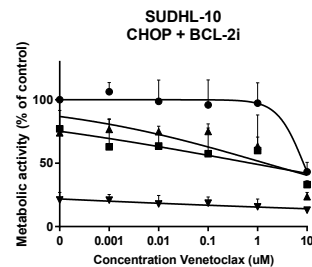**B**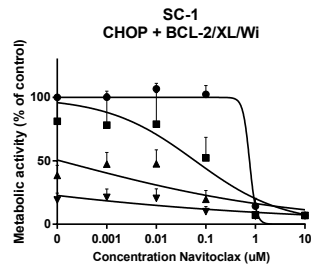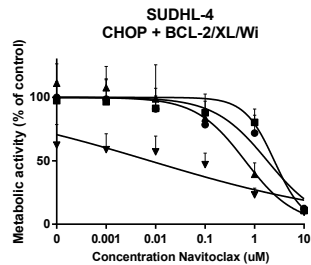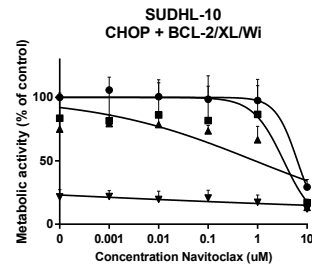**C**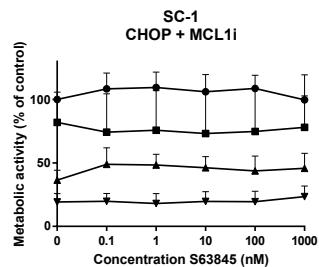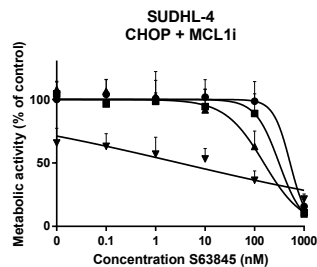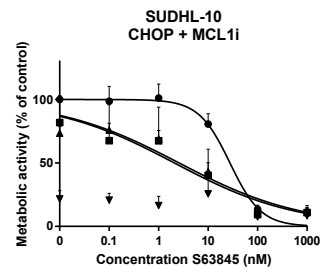

Supplement: Supplementary file 1 [file ijms-20-06036-s001.zip › SupplementalFigure5.pdf]

OCI-LY3

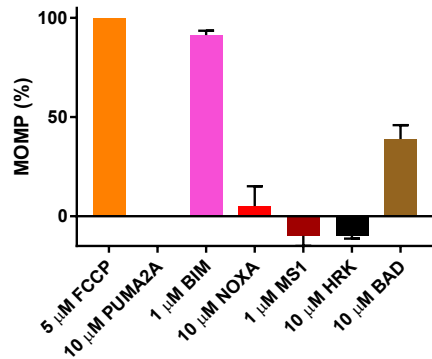

U-2932

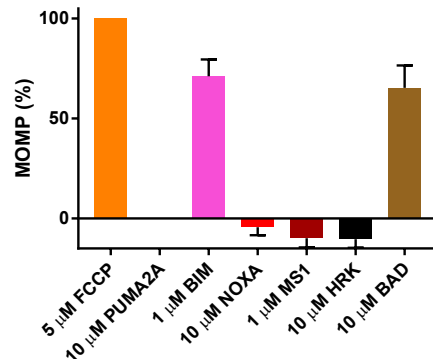

SUDHL-2

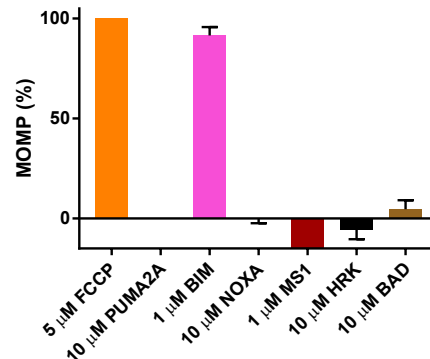

SUDHL-4

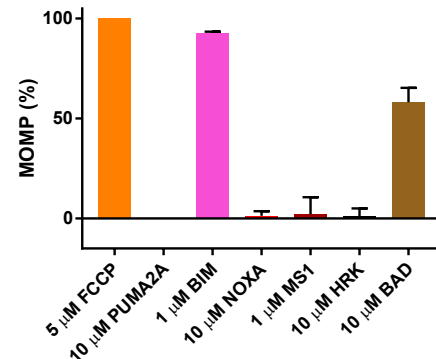

SUDHL-5

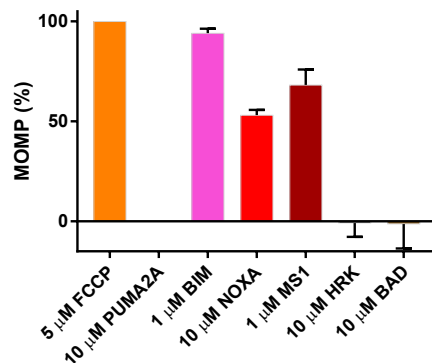

SUDHL-6

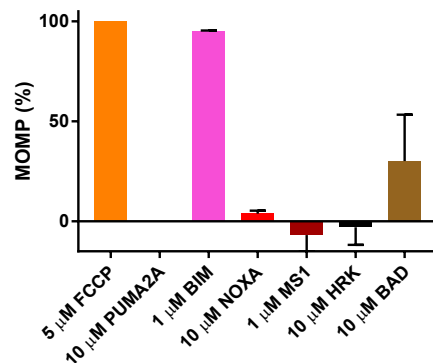

SUDHL-10

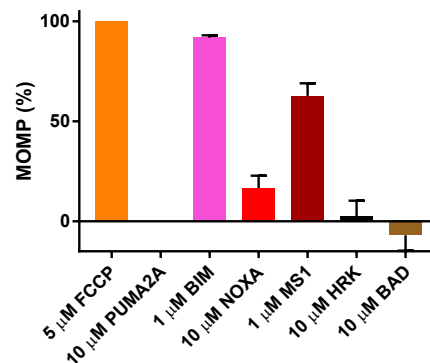

SC-1

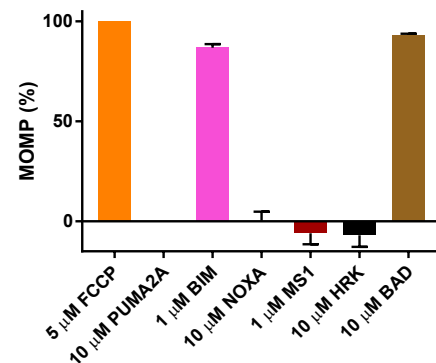

Supplement: Supplementary file 1 [file ijms-20-06036-s001.zip › SupplementalFigure1.pdf]

**A**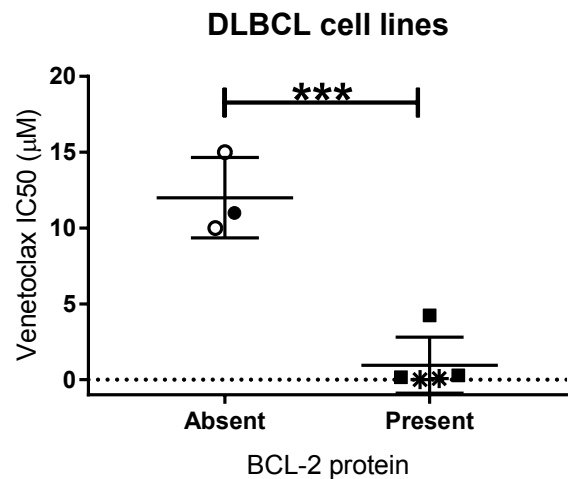**B**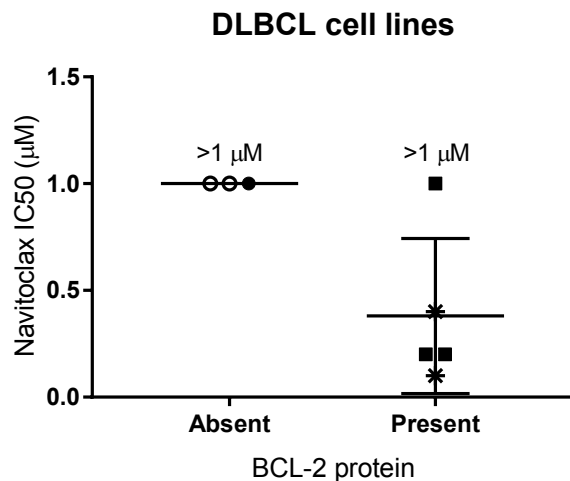**C**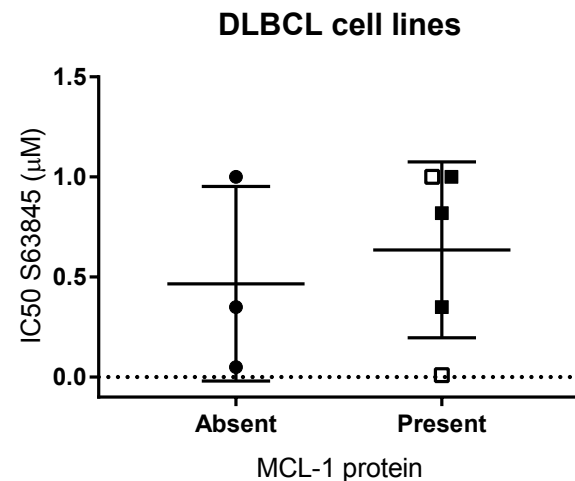**D**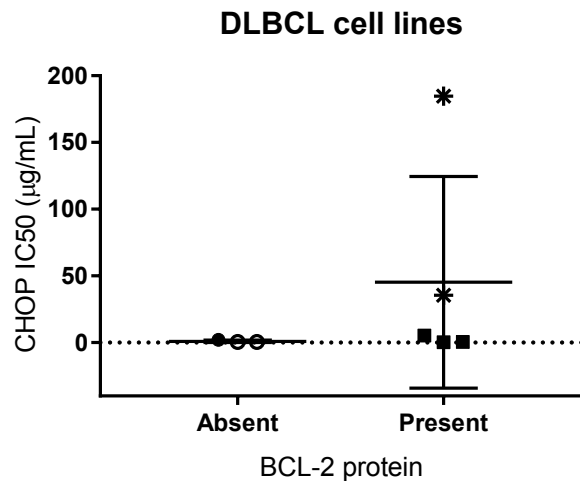**E**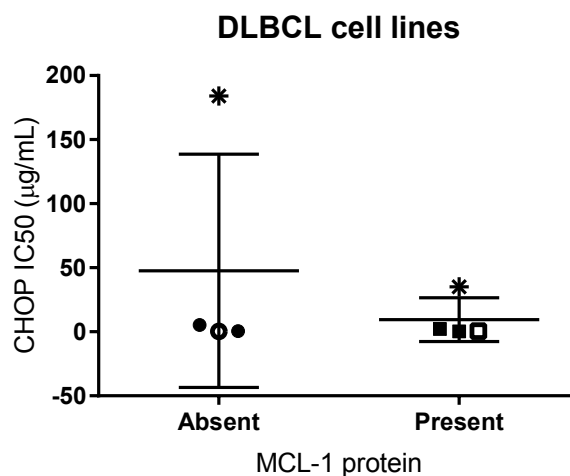

Supplement: Supplementary file 1 [file ijms-20-06036-s001.zip › SupplementalFigure3.pdf]

# BIM

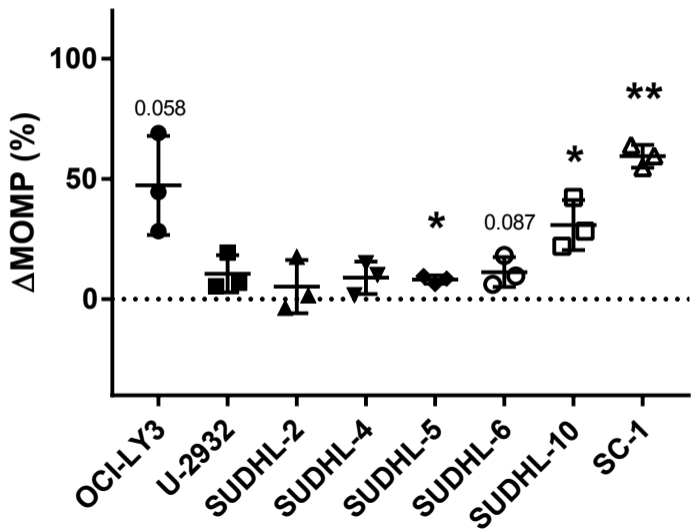

Supplement: Supplementary file 1 [file ijms-20-06036-s001.zip › SupplementalFigure4.pdf]
